# An Azimuth Antenna Pattern Estimation Method Based on Doppler Spectrum in SAR Ocean Images

**DOI:** 10.3390/s18041081

**Published:** 2018-04-03

**Authors:** Hui Meng, Xiaoqing Wang, Jinsong Chong

**Affiliations:** 1 National Key Laboratory of Science and Technology on Microwave Imaging, Beijing 100190, China; menghui14@mails.ucas.ac.cn; 2 Institute of Electronics, Chinese Academy of Sciences, Beijing 100190, China; 3 School of Electronics, Electrical and Communication Engineering, University of Chinese Academy of Sciences, Beijing 100190, China; 4 Institute of Microelectronics of Chinese Academy of Sciences, Beijing 100029, China

**Keywords:** azimuth antenna pattern, Doppler spectrum, low-scattering region, SAR Ocean images

## Abstract

In synthetic aperture radar (SAR) ocean remote sensing, it is very difficult to estimate an accurate azimuth antenna pattern (AAP) from low-scattering SAR images without strong scattering targets. Therefore, an azimuth antenna pattern estimation method based on Doppler spectrum in SAR ocean images is proposed in this paper. In order to preserve the complete AAP information, an azimuth unweighted matched filter is used to re-image the SAR raw data in the proposed method. Then, the shape factor of AAP can be obtained by linear statistics of the relationship between Doppler center and edge frequency spectrum in Doppler spectrum of each distance gate. In addition, the impact of the uniformity and signal-to-noise ratio of SAR ocean images on the estimation results are also analyzed by simulation. Finally, the feasibility of proposed method is verified by data from ERS-2 (European remote sensing satellite (ERS) was the European Space Agency’s first Earth-observing satellite). Experimental results show that the AAP estimated by proposed method has a good estimation result.

## 1. Introduction

In synthetic aperture radar (SAR) images, the accurate knowledge of the antenna patterns is of great significance for precise image processing and calibration [1]. Currently, whether airborne or spaceborne SAR, azimuth antenna pattern (AAP) monitoring is all mainly performed by exploiting transponders [2], which are quite expensive to be deployed and maintained. For the current spaceborne SAR, the antenna pattern changes little and does not require real-time calibration. For airborne SAR, it is necessary to perform a re-calibration test in every flight experiment, which makes it very important to measure the real-time values of the AAP. Therefore, in order to reduce the complexity and cost of the antenna calibration, it is worthwhile to extract the accurate information of the antenna pattern from SAR images.

Currently, several methods have been proposed for estimating the AAP from SAR images. For example, in 2003, Guarnieri, A.M. et al. [3,4] proposed a method to estimate the AAP from a strong point target in SAR images. In 2007, Tan, H. et al. [5] proposed a new simulated method to estimate the AAP using a strong point target. In this paper, the two-way AAP is estimated by applying the short-time Fourier transform to the original echo of the strong target. From 2011 to 2014, Guccione, P. et al. [6,7,8] proposed a method to estimate the AAP using the persistent strong point scatterers. The targets they use in this method have instead the following two features: (1) multiple images need to be collected in the same area; (2) the strong scattering targets contained in the image should have sparsity and time stability.

The above methods of estimating AAP from SAR images all have one thing in common: that is, the SAR image used to estimate the antenna pattern must contain at least one strong scattering point target with time stability. However, when we estimate the AAP from SAR ocean images, there is usually no strong scattering target with time stability. Therefore, the methods above cannot work.

In order to estimate the AAP more conveniently from ocean SAR images, an AAP estimation method based on Doppler spectrum in SAR ocean images is proposed in this paper. The procedure of the proposed method is as follows. Firstly, the relationship between the AAP and the Doppler spectrum is deduced by analyzing the composition of the Doppler spectrum of the SAR images. Secondly, the proper model parameters are estimated via the relationship between Doppler center spectrum and edge spectrum. Finally, the real value of the AAP is calculated by substituting the estimated AAP parameters into the model. In addition, the accuracy of the estimated AAP is indirectly verified by comparing the real Doppler spectrum with the Doppler spectrum calculated by the estimated AAP.

The rest of this paper is organized as follows. Section 2 gives the details of the methods and principles used in this method. In Section 3, simulation analysis of the applicability of the proposed method are presented. In Section 4, two real SAR data are used as examples for experimental validation. Finally, conclusions are presented in Section 5.

## 2. Azimuth Antenna Pattern Estimation Method Based on Doppler Spectrum

### 2.1. The Principle of the Proposed Method

#### 2.1.1. Analysis of Doppler Spectrum Composition

From the SAR imaging theory, it is well known that the shape of system noise, azimuthal ambiguity, and the backscattering signal present different patterns in the Doppler spectrum of the SAR raw signal (here, it is supposed that the range match filtering and range cell migration correction have been done), i.e., the system noise power density is a certain constant in the Doppler spectrum, whereas the shape of the Doppler spectrum of the backscattering signal and azimuthal ambiguity depend on the antenna pattern: the backscattering signal and azimuthal ambiguity correspond to the main lobe and side lobe respectively. Therefore, the Doppler spectrum of the SAR raw signal can be expressed as [9]

(1) $$E\left[p\left(f,x_{0},y_{0}\right)\right]=\sum_{n=-\infty }^{n=\infty }\bar{\sigma }\left(x_{0}+nD_{x},y_{0}+nD_{y}\right)P_{a}\left(f-f_{0}+nF_{r}\right)+\frac{N_{0}}{F_{r}}$$

where $\left[x_{0},y_{0}\right]$ are the center positions of the area upon which the Fourier transformation is applied; x and y are the coordinates in the flight and look directions respectively, E[·] refers to the mathematic expectation, f denotes the Doppler frequency, and p(f) denotes the azimuth power spectrum of the SAR raw signal. $P_{a}\left(f\right)$ is the power spectrum function of an ideal point target with a 0 dB normalized radar cross-section (NRCS), its shape is determined by the two-way AAP. Further, $f_{0}$ is the Doppler centroid, $F_{r}$ refers to the pulse repeat frequency of the SAR system, $N_{0}$ is the intrinsic noise floor of the SAR system, $\bar{\sigma }\left(x_{0}+nD_{x},y_{0}+nD_{y}\right)$ is the mean NRCS of the pixels located between $\left[x_{0}+nD_{x}-L/2,y_{0}+nD_{y}\right]$ and $\left[x_{0}+nD_{x}+L/2,y_{0}+nD_{y}\right]$ (L is the data length for calculating the Doppler spectrum), $D_{x}$ and $D_{y}$ are the displacements between the position of the azimuth ambiguity signal and the real target position in the flight and look directions, respectively. They can be written as [10]

(2) $$D_{x}=\frac{R\lambda F_{r}}{2V},D_{y}=-\frac{\lambda^{2}f_{0}F_{r}R}{4V^{2}}$$

where R is the slant range of the target, λ is the radar wavelength, and V is the velocity of the SAR platform.

Generally, in Equation (1), among the azimuthal ambiguity signals, only the first azimuth antenna side-lobes, which correspond to *n* = −1 and 1, are strong enough that cannot be omitted [9]. Hence, Equation (1) can be simplified as

(3) $$\begin{array}{l} E\left[p\left(f,x_{0},y_{0}\right)\right]\approx \bar{\sigma }\left(x_{0},y_{0}\right)P_{a}\left(f-f_{0}\right)+\bar{\sigma }\left(x_{0}+D_{x},y_{0}+D_{y}\right)P_{a}\left(f-f_{0}+F_{r}\right)\\ +\bar{\sigma }\left(x_{0}-D_{x},y_{0}-D_{y}\right)P_{a}\left(f-f_{0}-F_{r}\right)+\frac{N_{0}}{F_{r}} \end{array}$$

Equation (3) indicates that the shape of the averaged power spectrum of backscattering signal, azimuth ambiguity and system noise are determined by the AAP ($P_{a}\left(f\right)$) and $N_{0}$.

#### 2.1.2. Methods and Solutions to Estimate the Antenna Pattern from the Doppler Spectrum

In general, $D_{y}\ll D_{x}$ If the data length for calculating the Doppler spectrum is much larger than $D_{x}$ (for example, $L\gg D_{x}$) and SAR image scattering intensity in the observation area is relatively uniform, the $\bar{\sigma }\left(x_{0}+D_{x},y_{0}+D_{y}\right)$ and $\bar{\sigma }\left(x_{0}-D_{x},y_{0}-D_{y}\right)$ can be approximated by

(4) $$\bar{\sigma }\left(x_{0}+D_{x},y_{0}+D_{y}\right)\approx \bar{\sigma }\left(x_{0}-D_{x},y_{0}-D_{y}\right)\approx \bar{\sigma }\left(x_{0},y_{0}\right)$$

Hence, Equation (3) becomes

(5) $$E\left[p\left(f\right)\right]\approx \bar{\sigma }\left(x_{0}\right)\left[P_{a}\left(f-f_{0}\right)+P_{a}\left(f-f_{0}-F_{r}\right)+P_{a}\left(f-f_{0}+F_{r}\right)\right]+\frac{N_{0}}{F_{r}}$$

where $N_{0}/F_{r}$ is the average of the noise. Here, Equation (5) has been sufficiently averaged. Then, on the basis of Formula (5), the derivation process of estimating the antenna pattern from the SAR image is as follows.

$P_{a}\left(f\right)$ can have multiple unknown parameters. Therefore, we assume that the two-way AAP is expressed as

(6) $$P_{a}(f)=P_{a}(b_{1},b_{2},\ldots ,b_{m},f)$$

where $b_{1},b_{2},b_{3},\ldots ,b_{m}$ represent the parameters of AAP, m is the number of parameter.

However, in general, the number of the AAP parameters are limited. The Gaussian model [11] or the ${\mathrm{sinc}}^{4}$ model [3,7,8] is chosen as the AAP model. The ideal antenna pattern power spectrum is the ${\mathrm{sinc}}^{4}$ model (for example, ${\mathrm{sinc}}^{4}\left(\frac{L_{ant}}{2v}f\right)$, $L_{ant}$ is the antenna length) [7], especially for satellites such as ERS2 (European remote sensing satellite (ERS) was the European Space Agency’s first Earth-observing satellite), RADARSAT (RADARSAT is a Canadian remote sensing Earth observation satellite program overseen by the Canadian Space Agency), Envisat (Environmental Satellite) and most of the airborne SAR. Therefore, the applicability of this method is to estimate the AAP using the one parameter model as an example (in Appendix A, the method of estimating multi-parameter AAP will be given). The following section will show how to estimate the AAP from a one-parameter estimation model.

Since we are studying a single-parameter AAP estimation method, let *m* = 1. It is found through observation that Equation (5) can be simplified as an equation containing only two unknowns, AAP and system noise. therefore, the Equation (5) can be simplified by choosing two different frequency points in the Doppler spectrum (for example, $f_{1}$ and $f_{2}$), then the following linear relationship is derived out.

(7) $$E\left[p\left(f_{2}\right)\right]=\left\{E\left[p\left(f_{1}\right)\right]-E\left[p\left(f_{2}\right)\right]\right\}\alpha +\frac{N_{0}}{F_{r}}$$

where

(8) $$\alpha =\frac{P_{a}\left(f_{2}-f_{0}\right)+P_{a}\left(f_{2}-f_{0}-F_{r}\right)+P_{a}\left(f_{2}-f_{0}+F_{r}\right)}{P_{a}\left(f_{1}-f_{0}\right)+P_{a}\left(f_{1}-f_{0}-F_{r}\right)+P_{a}\left(f_{1}-f_{0}+F_{r}\right)-P_{a}\left(f_{2}-f_{0}\right)-P_{a}\left(f_{2}-f_{0}-F_{r}\right)-P_{a}\left(f_{2}-f_{0}+F_{r}\right)}$$

Equation (7) indicates that the relationship between $E\left[p\left(f_{2}\right)\right]$ and $E\left[p\left(f_{1}\right)\right]-E\left[p\left(f_{2}\right)\right]$ is linear, in which the constant term depends on the noise floor $N_{0}$, and the linear coefficient α depends on $P_{a}\left(f\right)$ [see Equation (8)]. Theoretically, if there are more than two sufficiently averaged Doppler spectra, α and $N_{0}$ can be resolved by equation system (7). More Doppler spectra will result in a more precise estimation of α and $N_{0}$. To increase the estimation precision, $f_{1}$ and $f_{2}$ should be selected to make $E\left[p\left(f_{1}\right)\right]-E\left[p\left(f_{2}\right)\right]$ as large as possible while $E\left[p\left(f_{2}\right)\right]$ as small as possible (in Appendix B, the influence of Doppler frequency point selection on AAP estimation accuracy is analyzed: that is why $f_{1}=f_{0}$ and $f_{2}=f_{0}-F_{r}/2$ are selected in this paper). 

After estimating α, $P_{a}\left(f\right)$ can be obtained further. in order to simply the reading, the one parameter sinc model is selected to estimate the AAP. Therefore, the two-way AAP model chosen in this paper is shown as

(9) $$P_{a}\left(f\right)=a{\mathrm{sinc}}^{4}\left(\frac{f}{b}\right)$$

where, a can be solved by $\int_{-3PRF/2}^{3PRF/2}a{\mathrm{sinc}}^{4}\left(\frac{f}{b}\right)df=1$, $b=\frac{2v}{L_{ant}}$ is the scale factor [12], f is the Doppler frequency.

Substituting Equation (9) into Equation (8), Equation (8) becomes

(10) $$\alpha =\frac{2a{\mathrm{sinc}}^{4}\left(\frac{F_{r}}{2b}\right)+a{\mathrm{sinc}}^{4}\left(\frac{3F_{r}}{2b}\right)}{1+2a{\mathrm{sinc}}^{4}\left(\frac{F_{r}}{b}\right)-2a{\mathrm{sinc}}^{4}\left(\frac{F_{r}}{2b}\right)-a{\mathrm{sinc}}^{4}\left(\frac{3F_{r}}{2b}\right)}$$

where α is the slope of the Equation (7), the right side of Equation (10) is a unary function of *b*. In general, the *Fr*’s cover the range of: $0.9b<F_{r}<1.5b$ [12], in this case, the above function is monotonically increasing. Therefore, the factor *b* can be obtained from Equation (10) by dichotomy, and then model $P_{a}\left(f\right)$ can be obtained by Equation (9). At the same time, the one-way AAP can be calculated out.

Strictly speaking, $P_{a}\left(f\right)$ depends on the slant range of the target: for example, α is a function of the slant range. In fact, when the image scattering value distribution is homogeneous, the differences in α between different range cells can be omitted. In many cases (such as the second example in Section 4) the differences in α between different range cells cannot be omitted. Therefore, the entire image can be divided into subsets, then *α* and $P_{a}\left(f\right)$ can be estimated from the subset with homogeneous. 

### 2.2. Method Flow Chart and Summary

The azimuthal matching filters of the standard imaging algorithm of commercial SAR products are generally weighted filters, which does not satisfy the basic requirements of the proposed method (the AAP information of the single-look complex (SLC) image obtained after weighted filtering will be distorted). Therefore, in order to retain the complete AAP information, the following steps are taken: in the first step, in SAR imaging, an unweighted azimuthal matching filter must be applied to the SAR raw data, and the SLC image of SAR data is obtained. In the second step, the Doppler center frequency must be estimated from the SLC image. Then, the Doppler centroids of the SLC image can be shifted to the zero-frequency. In the last step, the Doppler spectrum is calculated once every *L* pixels in the azimuthal data of each distance gate of the new SLC image. Then, the azimuth Doppler spectrum obtained from each distance gate is averaged separately to obtain a new range-Doppler image. By the statistics of the relationship between the Doppler central frequency and the edge frequency in every distance gate, the linear factor α is obtained. Finally, the appropriate AAP model is chosen to estimate the AAP of the SAR image.

**Figure 1:**
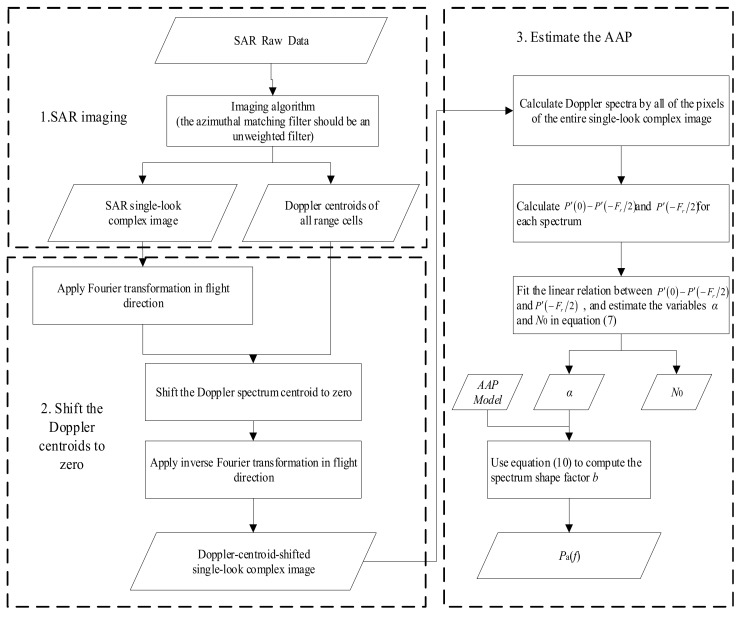
The flow chart of the proposed method. SAR: synthetic aperture radar; AAP: azimuth antenna pattern.

The proposed method is summarized in Figure 1.

## 3. Simulation Analysis of the Applicability of the Proposed Method

### 3.1. Parameters Settings of the Simulation

In order to satisfy the approximation of Equation (4), the ratio of the average NRCS of the ambiguous signal position to that of the true target position is chosen as 0.9 in this section. The simulation parameters are shown in Table 1.

### 3.2. Simulation Results

**Figure 2:**
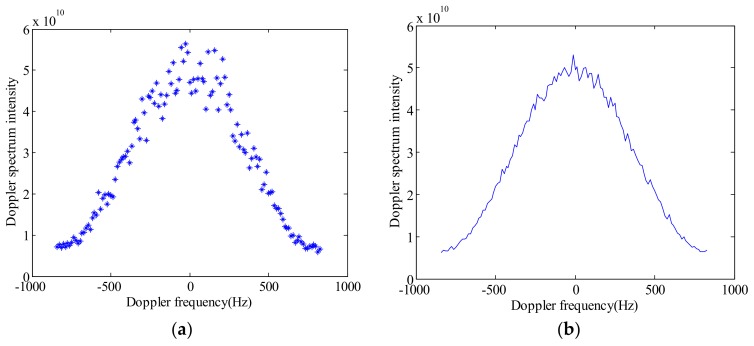
Simulated Doppler power spectrum. (**a**) Before multi-looking; (**b**) after multi-looking.

Based on the parameters in Table 1, we can generate simulation data of the Doppler power spectrum as shown in Figure 2.

**Figure 3:**
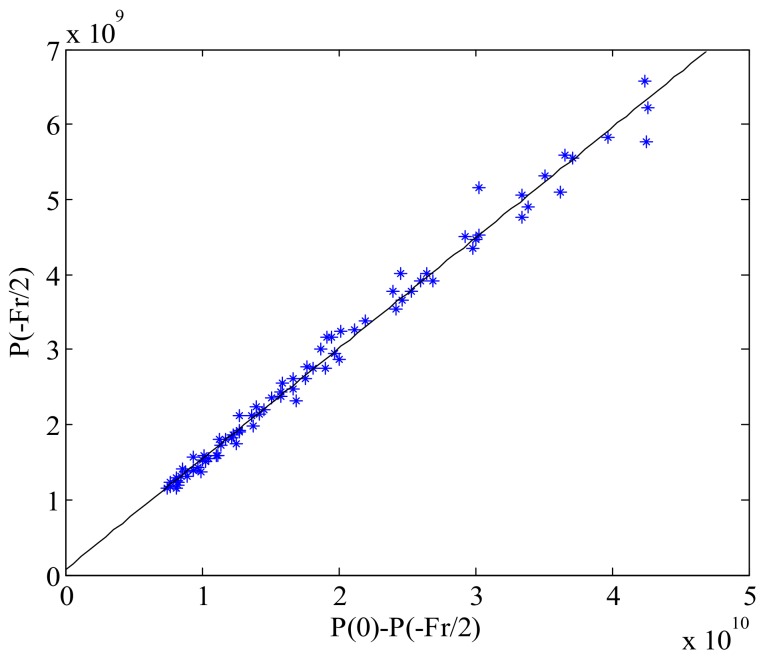
Relationship between $P\left(0\right)-P\left(-F_{r}/2\right)$ and $P\left(-F_{r}/2\right)$ for the simulated data.

We assume that the simulated power spectrum is P(f), and then use the same method to generate several Doppler power spectra to form a two-dimensional frequency domain image. The statistical relationship between $P\left(0\right)-P\left(-F_{r}/2\right)$ and $P\left(-F_{r}/2\right)$ from this simulated two-dimensional image is shown in Figure 3.

**Figure 4:**
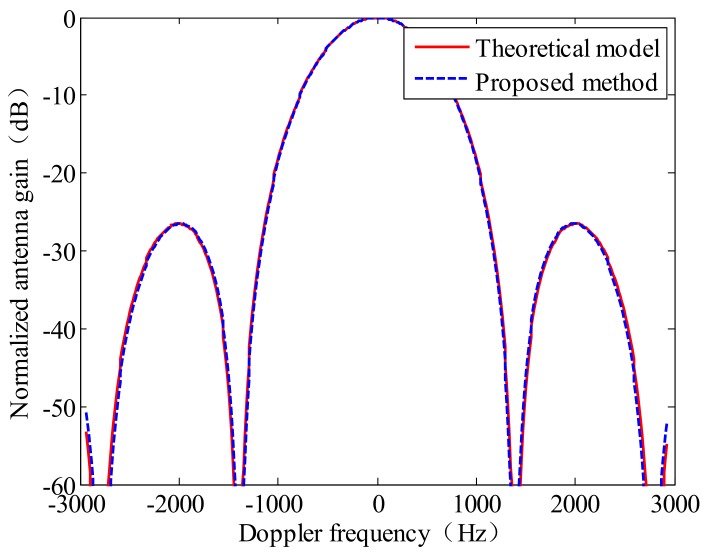
The comparison between the estimated two-way AAP and theoretical two-way AAP model.

From Figure 3, it can be seen that the relationship between $P\left(0\right)-P\left(-F_{r}/2\right)$ and $P\left(-F_{r}/2\right)$ is a linear relationship, approximately. This result validates the correctness of Equation (7). The variable α in Equation (7) is the slope of the fitted line in Figure 3. The scale factor *b* is calculated by the Equation (10), and then the AAP can be obtained by substituting *b* into Equation (9). The comparison between the estimated two-way AAP and theoretical two-way AAP model is shown in Figure 4. 

**Figure 5:**
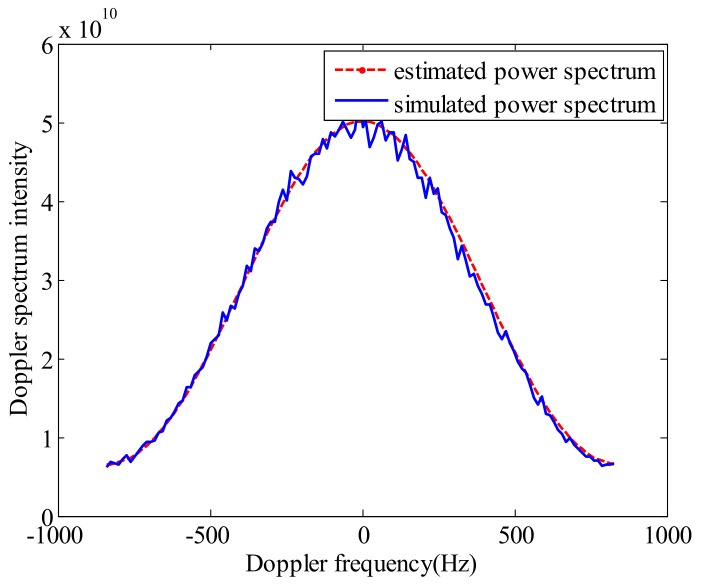
The comparison between the simulated power spectrum and estimated power spectrum.

In addition, by substituting the estimated AAP into Equation (3), the power spectrum can be obtained. The comparison chart between the estimated Doppler power spectrum and simulated one is shown in Figure 5.

Figure 5 shows that the estimated Doppler power spectrum is very close to the simulated original power spectrum. This result shows the feasibility of the proposed method.

### 3.3. Performance Analysis

To further confirm the effectiveness of the proposed method, 800 random Monte Carlo experiments are performed. The mean and the root mean square error (RMSE) of the estimated results are shown in Table 2.

From Table 2, it can be seen that the mean of the estimated value is very close to the real value, and that the RMSE of the parameter estimation achieved by the proposed method is rather low. The results show that the proposed method is very accurate.

### 3.4. Applicability Analysis of the Proposed Method

The influence of the uniformity of SAR image intensity and signal-to-noise ratio (SNR) on the estimation accuracy is mainly analyzed in this Section.

#### 3.4.1. The Influence of SAR Image Uniformity on Estimation results

In order to satisfy the approximation of Equation (4), the SAR ocean image intensity used to estimate the antenna pattern should be approximately uniform. Therefore, the influence of the intensity homogeneity of SAR marine images on the estimation results is analyzed in this section. 

Generally, SAR signals are affected by not only additional noise but also multiplicative speckle noise. As an example, we assume that the SAR signals can be described by a gamma distribution [13]. The probability distribution function of the SAR signal intensity can be expressed as

(11) $$f(I,\beta ,N)=\frac{\beta^{N}}{\Gamma (N)}I^{N-1}e^{-\beta I}$$

where I is the SAR signal intensity, β is the scale parameter, N is the number of multi-looking. The mean of the distribution function is μ=Nβ, and its standard deviation is $\sigma =\sqrt{N}\beta$. Therefore, $\sigma /\mu =1/\sqrt{N}$.

As we all know, for a SAR image, the greater the number of multi-looking, the smoother the image, and the stronger the image uniformity is. For ease of understanding, the impact of SAR image uniformity on the estimation results is simulated below. Among them, the uniformity of SAR images is mainly caused by the change of multi-looking.

According to Equation (11), the influence of the uniformity of SAR image intensity on the estimation results is simulated, the simulation parameters are shown in Table 3.

**Figure 6:**
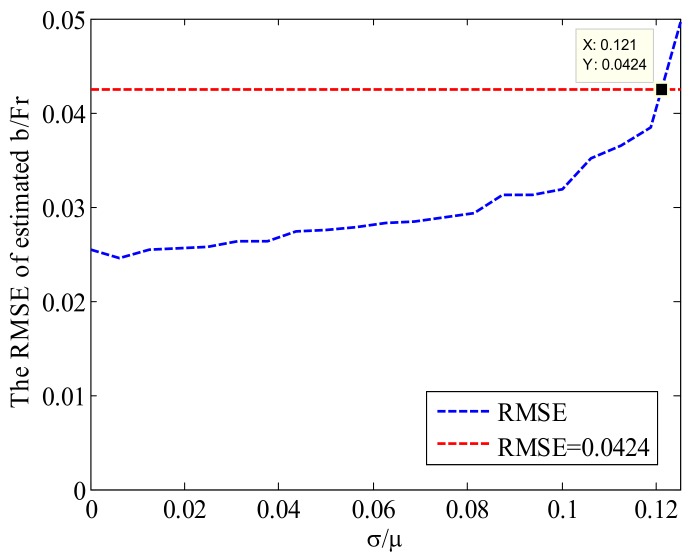
The RMSE of the estimated $b/F_{r}$ as a function of σ/μ.

The average of the scattering coefficient in Table 3 is a fixed value. When changing the value of σ/μ, the curve of the RMSE of the estimated $b/F_{r}$ is shown in Figure 6.

As can be seen from Figure 6, the larger the ratio of the variance of the image scattering coefficient to the mean value, the larger the error of the estimation value. The dashed red line in Figure 6 is the RMSE threshold calculated when the estimated error is equal to 5% of the true value. This means that when σ/μ is less than or equal to 0.121, the estimation result is considered to be more accurate. This means that the more uniform the SAR ocean image selected for estimating the antenna pattern, the closer the estimation results are to the real value.

#### 3.4.2. The Influence of the SNR of SAR Image on Estimation Results

The simulations were performed under different SNRs. The parameters of the simulations are given in Table 4.

**Figure 7:**
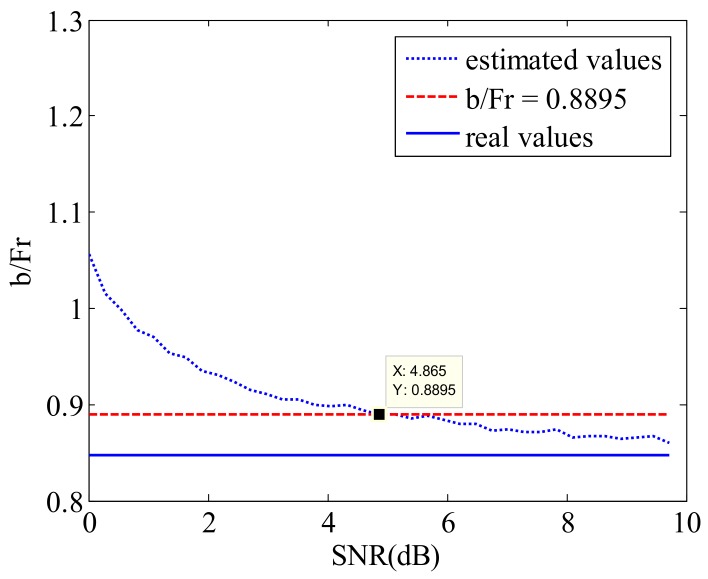
The comparison of estimated results and real values. The blue solid line is the real values of simulation data, the blue dotted line is the estimated values which is estimated by the proposed method, the red dashed line is the reference value ($b/F_{r}$ = 0.8895) which is 5% larger than the real values. The values between red dashed line and blue solid line are considered to be more accurate results. SNR: signal-to-noise ratio.

According to the parameters in Table 4, the effect of SNR on estimation accuracy is shown in Figure 7.

From Figure 7, it can be seen that with the increasing of SNR, the estimated values are close to real values. When the SNR is larger than 4.865 dB, the relative error between the estimated result and real value is less than 5%. Therefore, it can be considered that the estimated result reaches enough estimation accuracy.

## 4. Validation of the Proposed Method Based on Spaceborne SAR Data

In this section, two ERS-2 satellite SAR images will be used to estimate the AAP. The satellite parameters of ERS-2 are shown in Table 5.

The theoretical scale factor b can be calculated from the parameter values in 0, which is $0.849F_{r}$.

### 4.1. The SAR Ocean Images Preprocessing for Experimental Validation

**Figure 8:**
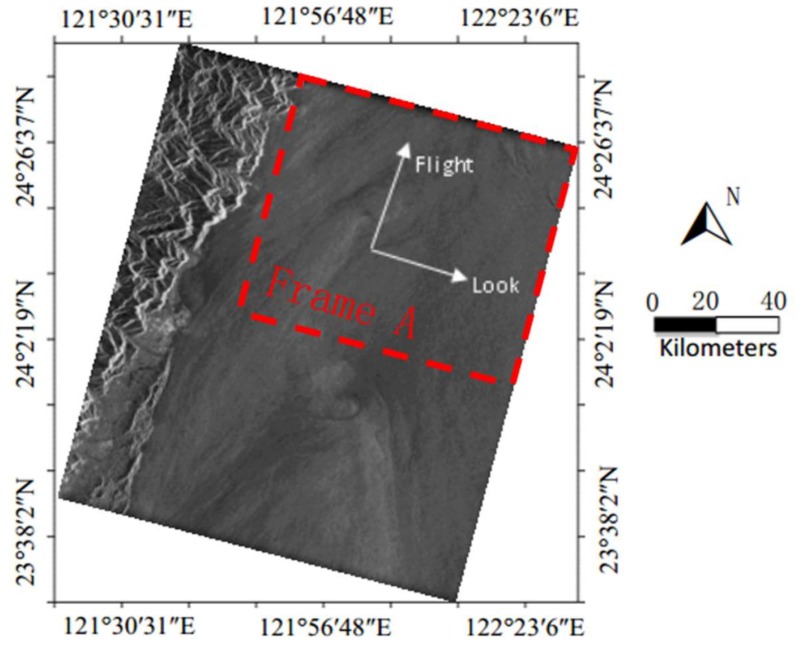
ERS-2 SAR image near the Taiwan in China collected on 9 June 2008, at 02:25 UTC.

Figure 8 is a SAR ocean image acquired by ERS-2 on 9 June 2008 near the Taiwan in China. There is a large area of uniform scattering SAR ocean image in Figure 8.

The red dashed frame, Frame A, in Figure 8 will be used for validation. There are 3450 pixels in the look direction and 14,336 pixels in the flight direction in the SLC image of Frame A. The procedure for estimating AAP from Frame A in Figure 8 is as follows.

The first step is to estimate the Doppler centroid $f_{0}$ for each range cell [12,14,15,16,17,18,19], and then shifting the Doppler spectrum centroid of the SLC image to zero. Although ocean currents can lead to an additional local shift of the Doppler centroid [19,20,21], however, the Doppler shift resulting from the ocean current is generally less than 5% of the pulse repetition frequency, which can be neglected in the method proposed in this paper. 

The second step is to calculate Doppler spectra from the SLC image. In this example, each Doppler spectrum is a 128-point discrete spectrum that is averaged by 224 times in the flight direction and 30 times in the look direction. Then, we can obtain 115 Doppler spectra. The azimuthal length used for calculating one Doppler spectrum is about 121 km (for example, *L* = 121 km). whereas $D_{x}$ is only about 5.67 km. In this case, *L* and $D_{x}$ satisfy the approximation condition of Equation (4) (for example, $L\gg D_{x}$).

**Figure 9:**
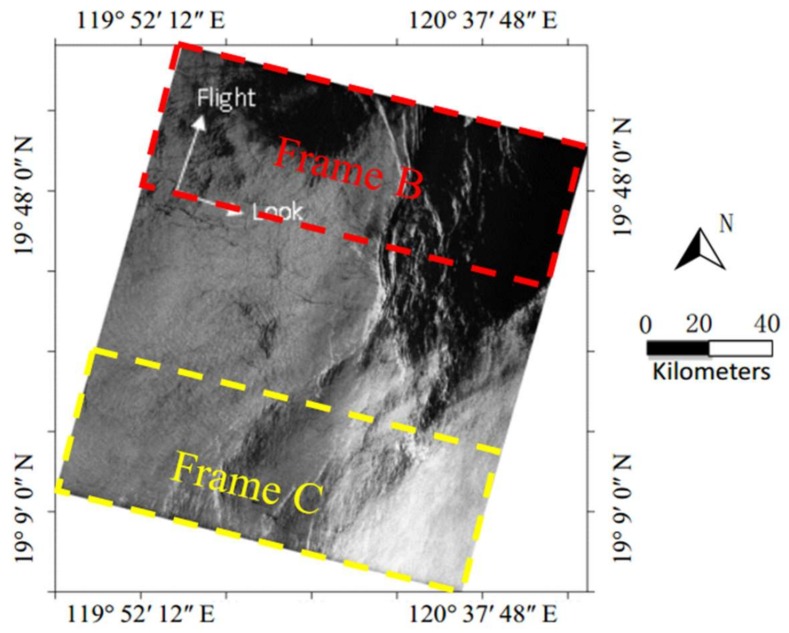
ERS-2 SAR ocean image of South China Sea collected on 30 April 2005, at 02:28 UTC.

Figure 9 is an ocean image acquired by ERS-2 on 30 April 2005 in the South China Sea.

There are 4912 pixels in the look direction and 28,695 pixels in the flight direction in the SLC image used in this Figure. From Figure 9, the SAR ocean image in this example contains some regions with poor uniformity, therefore, we select two regions as shown in Frame B and Frame C for verification and comparison. Frame B is an area with poor uniformity and Frame C is an area with strong uniformity. Then, the two areas will be further processed.

As in the first example, the first step is to estimate the Doppler centroid $f_{0}$ for each range cell, and then shift the Doppler spectrum centroid of the SLC image to zero frequency. The second step is to calculate Doppler spectra from the SLC image. Each Doppler spectrum in this example is also a 128-point discrete spectrum, which is averaged by 10 times in the flight direction and 70 times in the look direction. We can obtain 491 Doppler spectra from the SLC image in Frame B and Frame C, respectively. The azimuthal length used for calculating one Doppler spectrum also satisfy the approximation condition of Equation (4) (for example, $L\gg D_{x}$).

### 4.2. The Experimental Verification Results

**Figure 10:**
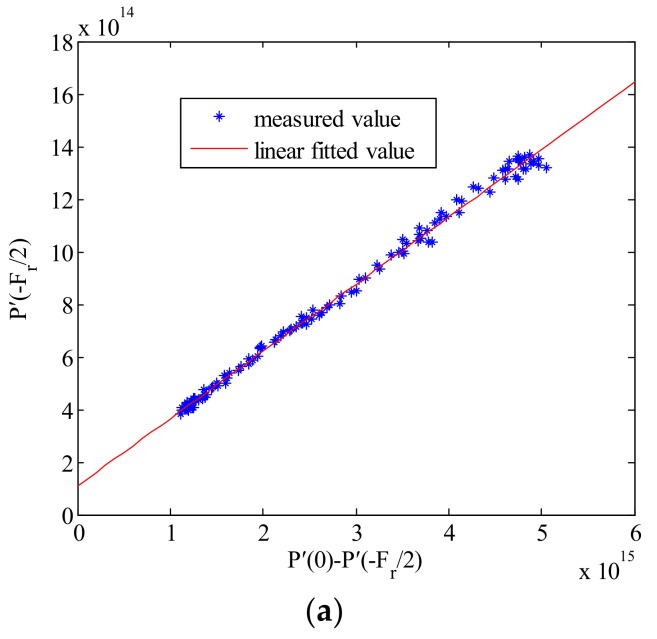

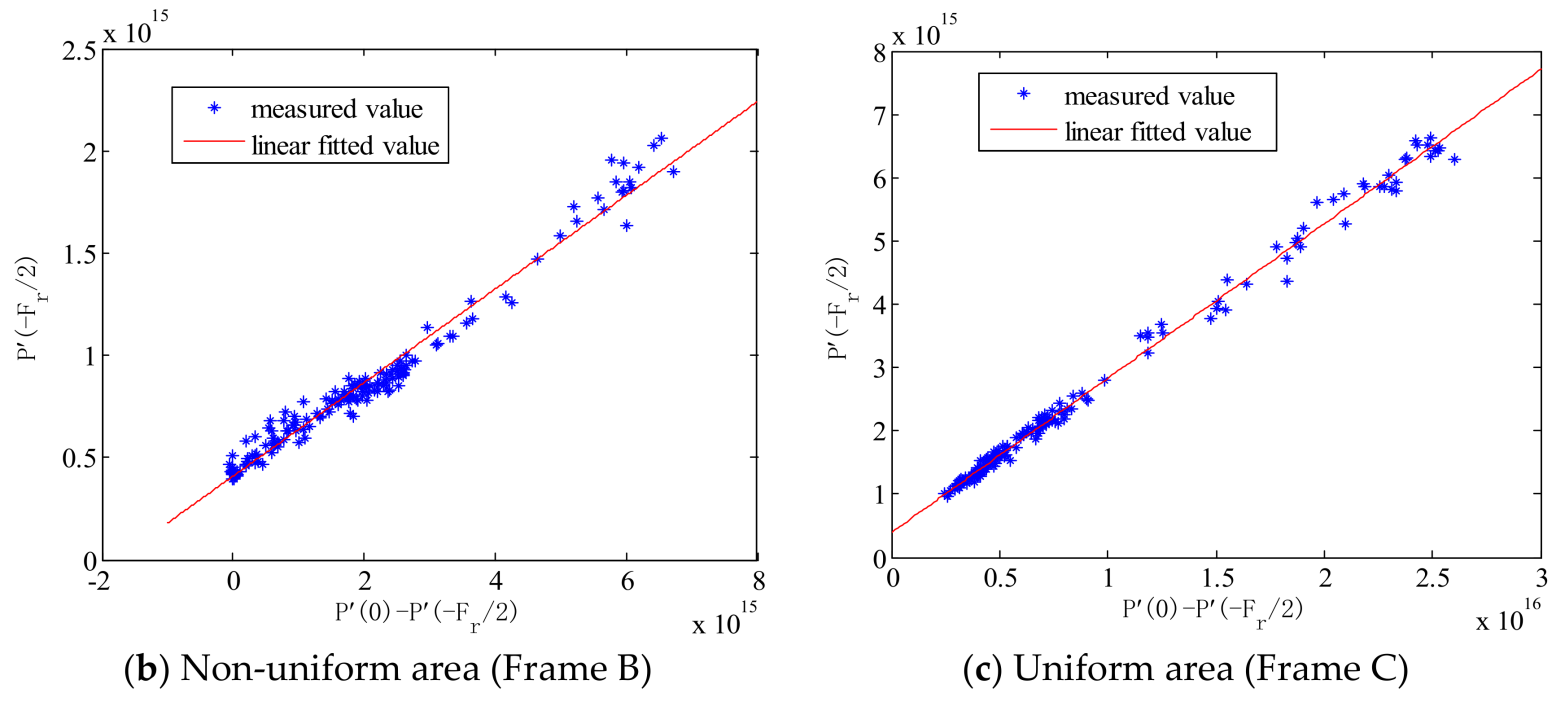
The comparison of statistical results between the uniform and non-uniform scattering regions in the second example. (**a**) Relationship between $P^{\prime }\left(0\right)-P^{\prime }\left(-F_{r}/2\right)$ and $P^{\prime }\left(-F_{r}/2\right)$ in Frame A, and the goodness of fit for the curve is 0.9994; (**b**) Relationship between $P^{\prime }\left(0\right)-P^{\prime }\left(-F_{r}/2\right)$ and $P^{\prime }\left(-F_{r}/2\right)$ in Frame B, and the goodness of fit for the curve is 0.9954; (**c**) Relationship between $P^{\prime }\left(0\right)-P^{\prime }\left(-F_{r}/2\right)$ and $P^{\prime }\left(-F_{r}/2\right)$ in Frame C, and the goodness of fit for the curve is 0.9984.

Denoting $P^{\prime }\left(f\right)$ as the shifted Doppler spectrum $P^{\prime }\left(f\right)=E\left[p\left(f+f_{0}\right)\right]$, the relationship between $P^{\prime }\left(0\right)-P^{\prime }\left(-F_{r}/2\right)$ and $P^{\prime }\left(-F_{r}/2\right)$ for Frame A, B and C are depicted in Figure 10.

From Figure 10, the statistical results of regions with non-uniform scattering (Frame B) are more scattered, whereas statistics of uniform regions (Frame A and Frame C) are closer to linear fitting curves. However, it can be seen that the relationship between $P^{\prime }\left(-F_{r}/2\right)$ and $P^{\prime }\left(0\right)-P^{\prime }\left(-F_{r}/2\right)$ is all very close to a linear function in those three areas.

After obtaining α, the scale factors $b=0.9F_{r}$, $b=0.93F_{r}$ and $b=0.91F_{r}$ can be obtained respectively from Frame A, Frame B and Frame C by solving Equation (10). 

In order to better reflect the estimation accuracy of the proposed method, the AAP characteristics are shown in Table 6 by comparing the measured values of Ottawa transponder [22], the estimated values of the proposed method, and the theoretical calculated values.

From Table 6, it can be concluded that the AAP obtained by transponder are very consistent with those estimated by the proposed method.

**Figure 11:**
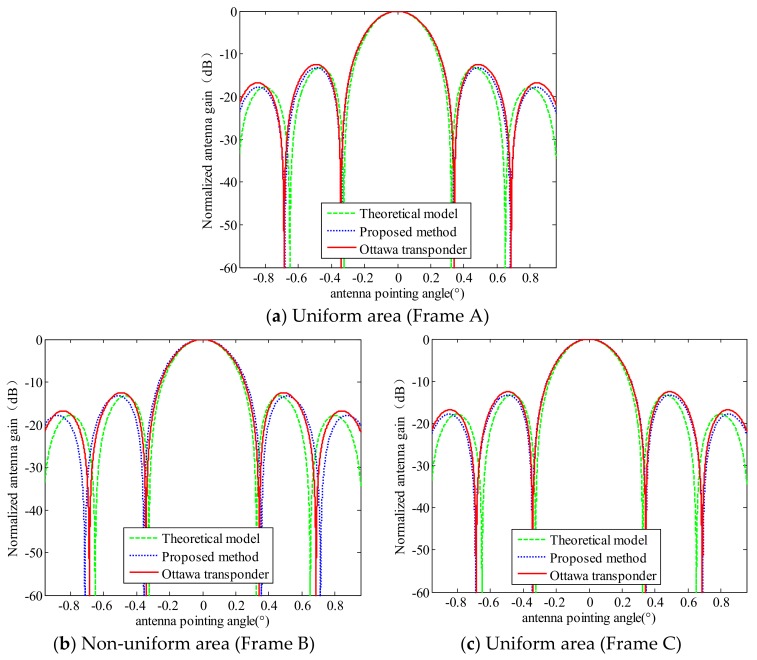
The comparison chart between the estimated AAP model, the result measured by Ottawa transponder and theoretical one-way model (${\mathrm{sinc}}^{2}(\frac{L_{a}}{2V}f)$, $L_{a}$ is the length of antenna). (**a**) The estimated AAP from Frame A; (**b**) the estimated AAP from Frame B; (**c**) the estimated AAP from Frame C.

The results of estimated AAP are compared with the result measured by Ottawa transponder, which is shown in Figure 11.

It is obvious to see from Figure 11 that the AAP estimated from Frame A and Frame C is closer to the result measured by Ottawa transponder than that estimated from Frame B. 

**Figure 12:**
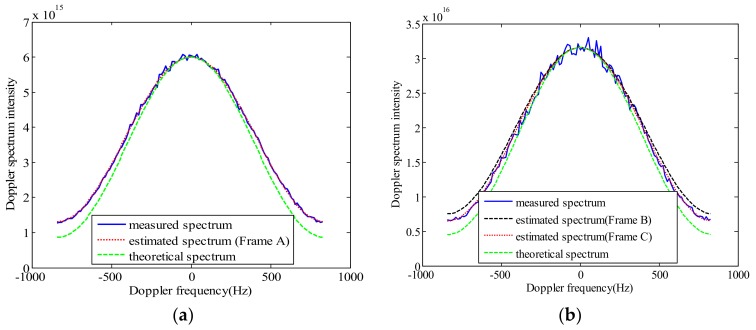
The comparison between the estimated spectrum, measured spectrum and theoretical spectrum. (**a**) Frame A in Figure 8; (**b**) Frame B and Frame C in Figure 9.

From the simulation data in Section 3, we also know that the proposed method has higher estimation accuracy. Therefore, from the analysis of Doppler spectrum in Figure 12, it can also be concluded that the estimated AAP model is more suitable for real SAR data than the theoretical AAP.

In Figure 12, the centroids of the measured and the estimated spectra are both shifted to zero. Figure 12 shows that the spectrum model estimated by the proposed method is superior to the theoretical spectrum model. In addition, the estimated spectrum of the uniform scattering region (Frame A and Frame C) is closer to the SAR real Doppler spectrum than that of the non-uniform region (Frame B). This shows the feasibility of the proposed method. At the same time, Figure 12 also shows that the ${\mathrm{sinc}}^{4}$ model is more suitable for the two-way AAP modeling of ERS-2 data.

In the above experiment, the number of pixels used to calculate the Doppler spectrum is chosen as 128, and which satisfy the approximate condition of Equation (4). When the number of pixels used to calculate the Doppler spectrum is changed, the estimation accuracy also will be changed. The results are shown in Table 7.

After the analysis of the results in Table 7, we can get the following conclusions: (1) The more uniform the selected image area, the higher the estimated accuracy is; (2) Under the same degree of uniformity, the more pixels used to calculate the Doppler frequency, the better the estimation effect.

## 5. Conclusions

In order to solve the problem that AAP cannot be estimated from low-scattering SAR images without strong scattering targets, an AAP estimation method based on the Doppler spectrum in SAR ocean images is proposed. The procedure is as follows. First of all, the region of the SAR ocean image with more uniform scattering intensity is selected. Then, the power spectrum of each distance gate can be calculated respectively. Secondly, two frequency points are selected from the power spectrum of each distance gate for mathematical statistics. Lastly, the shape factor of AAP is estimated based on the linear relationship of the statistical results, and then AAP is obtained. In addition, the accuracy of the estimated AAP is indirectly verified by comparing the real Doppler spectrum with the Doppler spectrum calculated by the estimated two-way AAP. At the same time, we also verify the accuracy of the one-way AAP estimated by the proposed method through comparison with transponder values.

In Appendix A, the estimation method of multi-parameter AAP is given. In Appendix B, the selection of two frequency points in the above estimation process is analyzed by the simulation, and the conclusion is that when the Doppler center frequency and the Doppler spectrum edge frequency are selected, the estimation result is the closest to the true value.

In order to analyze the practicability and feasibility of the proposed method, the influence of the uniformity of SAR image intensity and SNR on the estimation accuracy is simulated in this paper. After simulation, we conclude that the RMSE of the estimation results is larger when the uniformity of the SAR ocean image intensity is getting worse. When σ/μ = 1.21, the RMSE of the estimation result reaches the threshold, which is calculated when the estimated error is 5 percent of the true value. This threshold is limited to the conclusion which simulated by the Gamma distribution. In addition, the proposed method is also validated through simulation experiments and real SAR data. The verification results are in good agreement with the theoretical analysis. Meanwhile, when the influence of SNR on the estimation accuracy is simulated, the value when the estimated error is 5% of the true value is selected as the threshold. Therefore, we conclude that the proposed method can achieve good estimation results when the SNR is more than 4.865 dB; when the SNR is lower than 4.865 dB, the estimation error is larger.

However, the method we developed in this study is based on the condition that we have a known SAR AAP model. For an SAR image with unknown AAP model, the theory and method proposed in this paper need to be further improved. Therefore, more common AAP models are under study and will be part of the future work.

## Figures and Tables

**Table 1:** Parameters of the simulated data.

Parametric Name	Parametric Symbol	Parametric Value
Pulse repetition frequency	$F_{r}$	1679.902 (Hz)
Number of Doppler spectrum pixels	Azi_len	128
Simulation repeat number	Repeat_number	800
Look number of SAR image	M	10
Signal-to-Noise Ratio	SNR	5 dB
AAP scale factor	b	$0.849\times F_{r}$
Ratio of the scattering values of the blurred signal and the real target position	$\bar{\sigma }\left(x_{0}\pm D_{x},y_{0}\pm D_{y}\right)/\bar{\sigma }\left(x_{0},y_{0}\right)$	0.9

**Table 2:** The performance analysis of the proposed method.

AAP Parameters	Mean		RMSE
	Real Value	Estimated Value	
*b*/*F_r_*	0.849	0.843	0.025

**Table 3:** The simulation parameters in Figure 6.

	Values
The average of the scattering coefficient (μ)	$SNR\times N_{0}$
The standard deviation of the scattering coefficient (σ)	0∼0.12μ
Simulation repeat number	800

**Table 4:** Simulation parameters.

	Values
$\bar{\sigma }\left(x_{0},y_{0}\right)/N_{0}$	0–10 dB
$\bar{\sigma }\left(x_{0}\pm D_{x},y_{0}\pm D_{y}\right)/N_{0}$	$0.9\times \bar{\sigma }\left(x_{0},y_{0}\right)/N_{0}$
Simulation repeat number	800
Pixel number used for calculating one Doppler spectrum	80

**Table 5:** The related SAR parameters of the ERS-2.

Pulse Repetition Frequency ($F_{r}$)	Platform Velocity (V) (m/s)	Antenna Length $L_{a}$ (m)	Pulse Width (μs)	Bandwidth (MHz)	Wavelength (m)
1679.902	7131.7	10	37.12	15.5478	0.0566

**Table 6:** The AAP characteristics measured with Ottawa transponder, the estimated values and the theoretical values.

	Frame A			Frame B			Frame C		
	Mainlobe Width (°)	PSLR(Peak Sidelobe Ratio) (dB)	ISLR (Integral Sidelobe Ratio) (dB)	Mainlobe Width (°)	PSLR (dB)	ISLR (dB)	Mainlobe Width (°)	PSLR (dB)	ISLR (dB)
Transponder values	0.3030	−12.48	−10.27	0.3030	−12.48	−10.27	0.3030	−12.48	−10.27
Estimated values	0.3014	−13.26	−10.27	0.3150	−13.26	−10.28	0.3048	−13.26	−10.27
Theoretical values	0.2874	−13.26	−10.28	0.2874	−13.26	−10.28	0.2874	−13.26	−10.28

**Table 7:** The effect of the number of pixels used to calculate the Doppler spectrum on the estimation results of $b/F_{r}$.

Number of Pixels Used to Calculate the Doppler Spectrum	32	64	128
Frame B (Non-uniform area)	0.951	0.942	0.930
Frame C (Uniform area)	0.924	0.905	0.901

**Figure A1:**
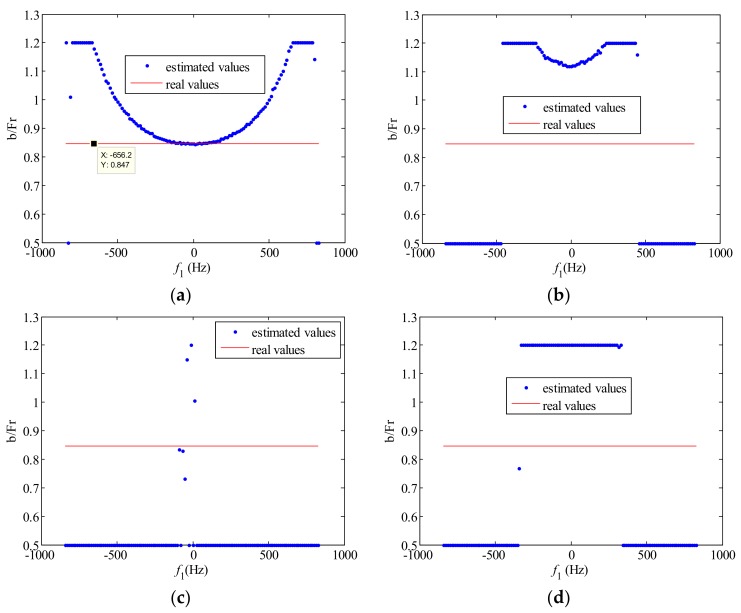

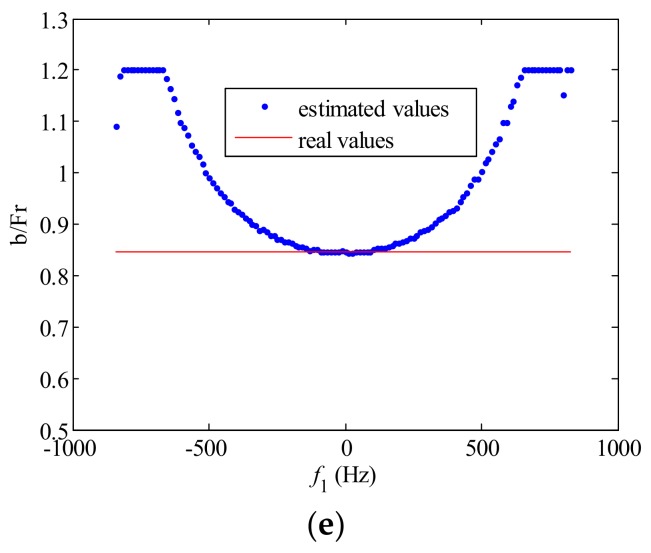
The statistical results of $b/F_{r}$, estimated by selecting different frequencies. (**a**) $f_{2}$ is the first frequency point, which represents the left edge of the Doppler spectrum; (**b**) $f_{2}$ is the 30th frequency point; (**c**) $f_{2}$ is the 64th frequency point, which is the Doppler centroid frequency; (**d**) $f_{2}$ is the 90th frequency point; (**e**) $f_{2}$ is the 128th frequency point, which represents the right edge of the Doppler spectrum; $f_{1}$ is the whole spectrum in (**a**–**e**).

## Appendix A. Multi-Parameter AAP Estimation Method

In this appendix, a multi-parameter AAP estimation method for m≠1 in Equation (6) is given. In order to estimate the whole unknown parameters of AAP, we select m+1 number of different frequency points on the Doppler spectrum, for example, $f_{1}$, $f_{2}$, …, $f_{m}$, substituting them into Equation (5), the following equation can be derived

(A1) $$\left\{ \begin{array}{l} E\left[p\left(f_{2}\right)\right]=\left\{E\left[p\left(f_{1}\right)\right]-E\left[p\left(f_{2}\right)\right]\right\}\alpha_{1}+\frac{N_{0}}{F_{r}}\\ E\left[p\left(f_{3}\right)\right]=\left\{E\left[p\left(f_{1}\right)\right]-E\left[p\left(f_{3}\right)\right]\right\}\alpha_{2}+\frac{N_{0}}{F_{r}}\\ \vdots \\ \vdots \\ E\left[p\left(f_{m+1}\right)\right]=\left\{E\left[p\left(f_{1}\right)\right]-E\left[p\left(f_{m+1}\right)\right]\right\}\alpha_{m}+\frac{N_{0}}{F_{r}} \end{array}\right.$$

where

(A2) $$\left\{ \begin{array}{l} \alpha_{1}=\frac{P_{a}\left(f_{2}-f_{0}\right)+P_{a}\left(f_{2}-f_{0}-F_{r}\right)+P_{a}\left(f_{2}-f_{0}+F_{r}\right)}{P_{a}\left(f_{1}-f_{0}\right)+P_{a}\left(f_{1}-f_{0}-F_{r}\right)+P_{a}\left(f_{1}-f_{0}+F_{r}\right)-P_{a}\left(f_{2}-f_{0}\right)-P_{a}\left(f_{2}-f_{0}-F_{r}\right)-P_{a}\left(f_{2}-f_{0}+F_{r}\right)}\\ \alpha_{2}=\frac{P_{a}\left(f_{3}-f_{0}\right)+P_{a}\left(f_{3}-f_{0}-F_{r}\right)+P_{a}\left(f_{3}-f_{0}+F_{r}\right)}{P_{a}\left(f_{1}-f_{0}\right)+P_{a}\left(f_{1}-f_{0}-F_{r}\right)+P_{a}\left(f_{1}-f_{0}+F_{r}\right)-P_{a}\left(f_{3}-f_{0}\right)-P_{a}\left(f_{3}-f_{0}-F_{r}\right)-P_{a}\left(f_{3}-f_{0}+F_{r}\right)}\\ \vdots \\ \vdots \\ \alpha_{m}=\frac{P_{a}\left(f_{m+1}-f_{0}\right)+P_{a}\left(f_{m+1}-f_{0}-F_{r}\right)+P_{a}\left(f_{m+1}-f_{0}+F_{r}\right)}{P_{a}\left(f_{1}-f_{0}\right)+P_{a}\left(f_{1}-f_{0}-F_{r}\right)+P_{a}\left(f_{1}-f_{0}+F_{r}\right)-P_{a}\left(f_{m+1}-f_{0}\right)-P_{a}\left(f_{m+1}-f_{0}-F_{r}\right)-P_{a}\left(f_{m+1}-f_{0}+F_{r}\right)} \end{array}\right.$$

Equation (A1) indicates that the relationship between $E\left[p\left(f_{m+1}\right)\right]$ and $E\left[p\left(f_{1}\right)\right]-E\left[p\left(f_{m+1}\right)\right]$ is linear. This is consistent with the analysis of Equation (7). According to the analysis in Appendix B, $f_{1}$ in Equation (A1) is selected as Doppler center frequency ($f_{0}$) position, $f_{2}$, …, $f_{m+1}$ are selected as the Doppler spectrum edge position as far as possible.

Substituting Equation (6) into Equation (A2), Equation (A2) becomes

(A3) $$\left\{ \begin{array}{l} \alpha_{1}=\frac{P_{a}\left(b_{1},b_{2},\cdots b_{m},f_{2}-f_{0}\right)+P_{a}\left(b_{1},b_{2},\cdots b_{m},f_{2}-f_{0}-F_{r}\right)+P_{a}\left(b_{1},b_{2},\cdots b_{m},f_{2}-f_{0}+F_{r}\right)}{P_{a}\left(b_{1},b_{2},\cdots b_{m},f_{1}-f_{0}\right)+P_{a}\left(b_{1},b_{2},\cdots b_{m},f_{1}-f_{0}-F_{r}\right)+P_{a}\left(b_{1},b_{2},\cdots b_{m},f_{1}-f_{0}+F_{r}\right)-P_{a}\left(b_{1},b_{2},\cdots b_{m},f_{2}-f_{0}\right)-P_{a}\left(b_{1},b_{2},\cdots b_{m},f_{2}-f_{0}-F_{r}\right)-P_{a}\left(b_{1},b_{2},\cdots b_{m},f_{2}-f_{0}+F_{r}\right)}\\ \alpha_{2}=\frac{P_{a}\left(b_{1},b_{2},\cdots b_{m},f_{3}-f_{0}\right)+P_{a}\left(b_{1},b_{2},\cdots b_{m},f_{3}-f_{0}-F_{r}\right)+P_{a}\left(b_{1},b_{2},\cdots b_{m},f_{3}-f_{0}+F_{r}\right)}{P_{a}\left(b_{1},b_{2},\cdots b_{m},f_{1}-f_{0}\right)+P_{a}\left(b_{1},b_{2},\cdots b_{m},f_{1}-f_{0}-F_{r}\right)+P_{a}\left(b_{1},b_{2},\cdots b_{m},f_{1}-f_{0}+F_{r}\right)-P_{a}\left(b_{1},b_{2},\cdots b_{m},f_{3}-f_{0}\right)-P_{a}\left(b_{1},b_{2},\cdots b_{m},f_{3}-f_{0}-F_{r}\right)-P_{a}\left(b_{1},b_{2},\cdots b_{m},f_{3}-f_{0}+F_{r}\right)}\\ \vdots \\ \vdots \\ \alpha_{m}=\frac{P_{a}\left(b_{1},b_{2},\cdots b_{m},f_{m+1}-f_{0}\right)+P_{a}\left(b_{1},b_{2},\cdots b_{m},f_{m+1}-f_{0}-F_{r}\right)+P_{a}\left(b_{1},b_{2},\cdots b_{m},f_{m+1}-f_{0}+F_{r}\right)}{P_{a}\left(b_{1},b_{2},\cdots b_{m},f_{1}-f_{0}\right)+P_{a}\left(b_{1},b_{2},\cdots b_{m},f_{1}-f_{0}-F_{r}\right)+P_{a}\left(b_{1},b_{2},\cdots b_{m},f_{1}-f_{0}+F_{r}\right)-P_{a}\left(b_{1},b_{2},\cdots b_{m},f_{m+1}-f_{0}\right)-P_{a}\left(b_{1},b_{2},\cdots b_{m},f_{m+1}-f_{0}-F_{r}\right)-P_{a}\left(b_{1},b_{2},\cdots b_{m},f_{m+1}-f_{0}+F_{r}\right)} \end{array}\right.$$

To solve all the unknown variables from Equation (A3), the Newton iterative method can be adopted. Then, the AAP can be obtained when all the variables that have been solved are substituting into Equation (6).

## Appendix B. The Influence of Doppler Frequency Point Setting on AAP Estimation Accuracy

In this appendix, the influence of Doppler frequency point setting on AAP estimation accuracy will be verified by simulations. The linear relationship statistics of Equation (7) is made by selecting two frequency points ($f_{1}$ and $f_{2}$) in the Doppler spectrum. Among them, $f_{2}$ is chosen as the following five different frequency positions. They are the spectrum left edge (the first frequency point), the 30th frequency point, Doppler centroid (the 64th frequency point), the 90th frequency point and the spectrum right edge (the 128th frequency point). $f_{1}$ traverses the entire spectrum. The estimated results of $b/F_{r}$ are shown in a, b, c, d, e in Figure A1.

As we can see from Figure A1, the estimated accuracy of $b/F_{r}$ is the highest when $f_{2}$ is the spectrum edge and $f_{1}$ is close to the Doppler centroid.
